# The domestic chick as an animal model of autism spectrum disorder: building adaptive social perceptions through prenatally formed predispositions

**DOI:** 10.3389/fnins.2024.1279947

**Published:** 2024-01-31

**Authors:** Toshiya Matsushima, Takeshi Izumi, Giorgio Vallortigara

**Affiliations:** ^1^Department of Biology, Faculty of Science, Hokkaido University, Sapporo, Japan; ^2^Faculty of Pharmaceutical Science, Health Science University of Hokkaido, Tobetsu, Japan; ^3^Centre for Mind/Brain Sciences, University of Trento, Rovereto, Italy

**Keywords:** autism spectrum disorder, domestic chicks, imprinting, biological motion, face perception, valproic acid, neonicotinoids, bumetanide

## Abstract

Equipped with an early social predisposition immediately post-birth, humans typically form associations with mothers and other family members through exposure learning, canalized by a prenatally formed predisposition of visual preference to biological motion, face configuration, and other cues of animacy. If impaired, reduced preferences can lead to social interaction impairments such as autism spectrum disorder (ASD) via misguided canalization. Despite being taxonomically distant, domestic chicks could also follow a homologous developmental trajectory toward adaptive socialization through imprinting, which is guided via predisposed preferences similar to those of humans, thereby suggesting that chicks are a valid animal model of ASD. In addition to the phenotypic similarities in predisposition with human newborns, accumulating evidence on the responsible molecular mechanisms suggests the construct validity of the chick model. Considering the recent progress in the evo-devo studies in vertebrates, we reviewed the advantages and limitations of the chick model of developmental mental diseases in humans.

## 1 Introduction

### 1.1 In search of valid animal models of developmental psychiatric disorders

Biological psychiatry and comparative psychology share a scientific question as for whether the apparent neurocognitive similarities among animals of different taxa (mostly vertebrates) could stem from common (homological) origins, namely that the underlying brain-behavior linkage evolved just once (Striedter, 2015). Alternatively, similar traits could arise as analogy via convergent evolution after multiple and independent events in each clade, therefore the challenges to find valid animal model of psychiatric disease are inevitably unsuccessful. Actually, the nervous system of vertebrates has held a highly conserved ground plan or “Bauplan” since the Cambrian period (Grillner and Robertson, 2016; Suryanarayana et al., 2021a,b); homologies are widely found from the level of composite molecules (transmitters, hormones, receptors, and second messengers) to neural pathways and cytoarchitectonic organizations at the macroscopic level (Güntürkün and Bugnyar, 2016; Shepherd and Grillner, 2018) [specifically for the mammal-bird homologies, see Güntürkün and Bugnyar (2016)]. Despite the conservative nature of the organization of central nervous system (CNS), huge phenotypic diversification occurs in the behavior and cognition of vertebrates through differentiated development (Bateson and Gluckman, 2011), complicating the untangling of the evolutionary processes. By using a common set of composite parts and wirings in the brain, animals of different taxa could have developed diverse machineries for adaptive behaviors, so that the seemingly identical phenotypes are due to convergent evolution, hence the analogy.

In the present review, with the aid of recent progresses in evo-devo studies, we argue a possibility that chicks and humans exhibit a homologous developmental trajectory leading to similar predisposition in their visual perception. Here, we propose a working hypothesis that taxonomically distinct animals could develop social perception via common ontogenetic processes during the prenatal embryonic period and the subsequent early post-natal period. It is stressed that the early-life experiences are under strong control of embryonic development of the social perception in both species. Neural events during the late embryonic stages set up the neonatal predisposition such as face and biological motion preferences, so that the babies appear to be *“born knowing”* without specific post-natal experiences. Some of the late embryonic events could thus have lasting neurobehavioral effects on social communication throughout life. Specifically, as encouraged by recent progresses of avian model studies (Csillag et al., 2022; Huang and Cheng, 2022), validity of the domestic chicks as an autism spectrum disorder (ASD) model animal will be examined on two aspects, the surface and construct validity as criteria of the depression animal model proposed by Willner (1984). The former (surface validity) includes an examination of the similarity of behaviors and developmental processes, particularly the perceptual predisposition to face, biological motion and animacy. The latter (construct validity) includes shared neural substrates and molecular cascades, particularly the potential roles of nicotinic acetylcholine (nAChR) transmission in the embryonic brain in controlling excitation-inhibition balance in neonates. If the ASD-like impairments found in chicks are homologous to the human ASD, we will find these construct molecular events are based on identical gene expression profiles and cellular events during the prenatal period. If otherwise, and the impaired visual perception in chicks is due to convergent evolution, we will find differences in the relevant brain regions and/or the molecular events.

### 1.2 Diverse environmental risk factors of ASD remain to be specified

ASD is the most prevalent developmental disorder primarily characterized by underdeveloped social interactions and communication and restricted and repetitive patterns of behavior and interests (Wing and Gould, 1979; American Psychiatric Association, 2013; World Health Organization, 2023). It is speculated that the heterogeneous diagnostic phenotypes, as well as the dimensional nature of this disorder, could be associated with a wide range of underlying genetic and environmental factors (Tchaconas and Adesman, 2013). In addition to apparent genetic risks (Sandin et al., 2014) [for an exhaustive hereditability study in Sweden; also see Iossifov et al. (2014) for *de novo* mutations and Gaugler et al. (2014) for common variations], exposure to environmental toxicants during pregnancy and the neonatal period remains a major social and scientific concern (Rossignol and Frye, 2014). For example, a large-scale twin study (Hallmayer et al., 2011) revealed high concordance rates among siblings, indicative of the role of genetic factors. However, the authors also reported that common environmental factors shared by these twins could substantially contribute to autism/ASD liability. Complex interactions are thought to occur between the genetic background of ASD susceptibility and the chemical agents acting during pregnancy and the early post-natal period.

However, it does not mean that these chemicals must be just eliminated. For example, valproic acid (VPA) has been identified as a risk factor for ASD (Christensen et al., 2013), but it remains an indispensable antiepileptic medication (Meador and Loring, 2013). The associated risks must be precisely evaluated in close consideration with known benefits. Studies using appropriate animal models are critical (Ergaz et al., 2016) because environmental risk management is difficult without reliable neurobehavioral measures of the symptoms. As epidemiological studies of diseases and epidemiological analyses are complimentary, it is critically important to find appropriate animal models. Rodents (mice and rats) are the most popular model animals because developed genetic tools are available (Mabunga et al., 2015; Chaliha et al., 2020). However, it must be noticed that rodents and primates have undergone distinct evolution since their separation during the late Cretaceous period over 66 million years ago.

**Figure 1:**
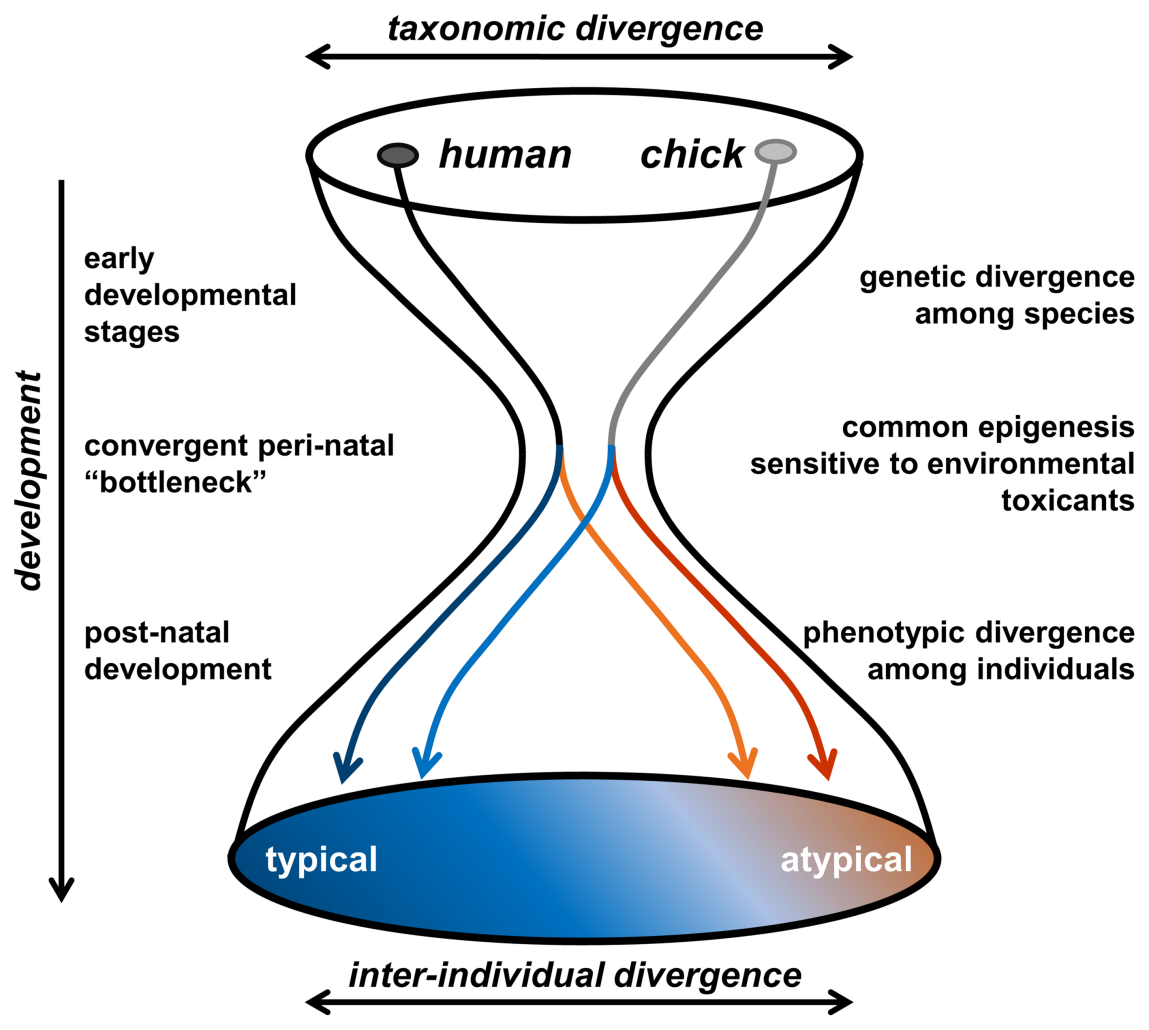
Hypothetical hourglass “bottleneck” model of the convergent evolution of peri-natal epigenetic control of social behaviors. During the early developmental stages, humans (primate) and chicks (birds) show a clear taxonomic divergence. Most organogenetic events are accomplished until the late embryonic period, namely before the third trimester in humans and the 14 days of incubation in domestic chicks. Convergent pre- and early post-natal “bottleneck” appears [i.e., phylotypic period (Irie and Kuratani, 2011)], and epigenetic processes (though mostly unspecified at present) would occur in the visual system responsible for the social perceptions. Subsequent developmental trajectory would be most sensitive to environmental toxicants during this “bottleneck” period, so that the inter-individual divergence overrides the taxonomic divergence.

As an alternative animal model, we have focused on newly hatched domesticated chicks (*Gallus gallus domesticus*) (Rosa-Salva et al., 2011; Vallortigara, 2012; Rosa-Salva et al., 2015; Versace et al., 2018). Birds are descendants of theropod dinosaurs, the major group of sauropsids, whereas primitive mammals diversified as a minor group of synapsids during the Carboniferous Period, ~ 300 million years ago. Considering such a taxonomically distinct animal as a valid model for human psychiatric disorders may sound unrealistic. If the adult phenotypes are solely compared, humans can never be chickens. However, as the hourglass bottleneck theory suggests (Irie and Kuratani, 2011), humans and chickens achieve developmental convergence during the prenatal/early neonatal periods in terms of specific neurocognitive aspects (Figure 1). It must be noticed that the hourglass model (Irie and Kuratani, 2011; Uesaka et al., 2019, 2020) has been proposed to explain the morphogenesis, rather than the CNS and the relevant behavioral traits. The phylotypical “bottleneck” period critical for the predisposition formation (namely, the responsive brain mechanisms) could therefore be different from that for the morphogenesis (i.e., mid-late embryonic stages). In order to determine whether certain animals are human-like in their perceptive natures, we must elucidate how phenotypic similarities arise through their respective development of the sensory system.

## 2 Surface validity

### 2.1 Visual predispositions canalize the development of social behaviors: common developmental features for the surface validity

It is speculated that the development of socialized behaviors during the early neonatal period depends on predisposed visual preference. In their inspiring report, Morton and Johnson (1991) argued that human babies are born to know visual characteristics of faces and denoted the information prior by term “CONSPEC.” With the aid of the “CONSPEC” information, neonates are supposed to gradually learn about the visual characteristics of conspecifics such as siblings and parents, and the device responsible for the subsequent learning to form social attachments was referred to as “CONLERN.” The predisposed “CONSPEC” information, or “core knowledge” (Spelke and Kinzler, 2007), could include a variety of visual features other than face (for reviews see Versace and Vallortigara, 2015; Di Giorgio et al., 2017; Lorenzi and Vallortigara, 2021; Rosa-Salva et al., 2021; Vallortigara, 2021a; Lemaire and Vallortigara, 2022), however, most studies have focused on face configuration, biological motion, and other cues of animacy.

#### 2.1.1 Face-like configuration

Differential tuning to face configuration has been demonstrated in adolescents with ASD when compared with typically developing controls (for one of the earliest reviews, see Johnson, 1992; Johnson, 2005; also see Pavlova et al., 2017 for a recent study using pictures resembling the food-face paintings by Giuseppe Arcimboldo). Based on the finding that infants prefer faces at 2–4 months old (Mauer and Barrera, 1981; Mondloch et al., 1999), a predisposed face preference has been suggested as a reliable diagnostic indicator for ASD. Typically, a definitive ASD diagnosis can be achieved in toddlers aged ~3 years; reliable biomarkers at earlier ages, if available, could facilitate treatment at the early stages (Elsabbagh and Johnson, 2009). In addition to behavioral measures, physiological measures, such as electroencephalography (EEG), can be employed in newborn babies (Buiatti et al., 2019).

Actually, the visual preference to face-like configuration could arise as a predisposed prior early in life. Visually naïve monkeys deprived of social stimuli (including human faces) preferred faces (Sugita, 2008), indicating robust development of face perception. Even human embryos in the third trimester of pregnancy reportedly exhibit head-turn responses to upright face configurations (Reid et al., 2017). A follow-up study confirmed the preference to face-like configuration, but top-heavy bias (i.e., inversion effect) was not detected (Reissland et al., 2020); it is reported also that the maternal mental health had significant effects on the embryonic activity, thus suggesting further studies to confirm the findings.

Subcortical visual pathway (superior colliculus/pulvinar to the amygdala) is responsible for the proto-face configuration (three blobs configured at a low spatial frequency) in human neonates (Johnson, 1992). This subcortical (or tectofugal) pathway is functional throughout life and plays critical role in rapid perception of emotional (e.g., fearful) face (Pegna et al., 2005; Tamietto and de Gelder, 2010); see Inagaki et al. (2022) for the most reliable account of this pathway in adult rhesus monkeys. Consistently, damage to the amygdala has been shown to cause some ASD-like deficiencies according to the “theory of mind” [ToM (Stone et al., 2003)], although the causal link between the ToM and ASD remains controversial (Gernsbacher and Yergeau, 2019).

Considering that the subcortical face pathway is functional in the early prenatal period and critical for the typical development of social behavior (Baron-Cohen et al., 2000), we would expect to detect differentiated visual attention to the face in infants with familial risk of ASD, and more specifically, in those who are later diagnosed with ASD. However, the use of the “face pop-up” task in these infants revealed clear attention to face similar to that observed in control individuals (or even higher) at 7 and 14 months of age (Elsabbagh et al., 2013). Conversely, another longitudinal developmental study has reported a steady decline in selective eye-fixating behavior in infants who were subsequently diagnosed with ASD at the earlier developmental stage from 2 to 6 months of age (Jones and Klin, 2013). The predisposed face perception is not a fixed trait, and the developmental changes need to be carefully examined (Simion and Di Giorgio, 2015). More recently, it has been reported that newborns with a high familial risk of ASD (6–10 days of age) show a reduced face preference when compared with low-risk controls (Di Giorgio et al., 2016a); also see Di Giorgio et al. (2021a) for the follow-up longitudinal study on 4-month-old infants.

Visually naïve newly hatched chicks also exhibit an evident inborn preference for faces (Rosa-Salva et al., 2010, 2011, 2019). Exposure to VPA during the late embryonic stage substantially reduced the face preference score (Adiletta et al., 2021), possibly paralleling that observed in humans (Di Giorgio et al., 2016a). The subcortical visual pathway of birds (optic tectum and arcopallium/nucleus taeniae amygdala) functionally mature early, and newly hatched chicks start actively pecking small conspicuous objects at 1–2 days old. Furthermore, immediate early gene imaging studies revealed that telencephalic limbic nuclei are involved in the predisposition preference of chicks (Mayer et al., 2016, 2017, 2019). Note that traditional rodent models fail to spontaneously exhibit a comparable visual predisposition. On the other hand, tortoise hatchlings show visual preference to face-like configuration over comparable alternatives without any preceding visual experience (Versace et al., 2020). As these tortoises are solitary species without parental care after hatching, we may assume that the social preference to face could be dated back to the common ancestor of amniotic vertebrates, naturally including birds and mammals. In other words, the predisposed preference may not be a high faculty requiring cortical computations.

Issues on the eye contact deficits in ASD needs a careful consideration. Human eyes comprise a unique tool for social communications due to their morphological feature (Kobayashi and Kohshima, 1997); the exposed white sclera surrounds the dark iris, making it easier for others to detect what the subject is looking. The “eye contact” tactics could therefore be unique to humans, despite the subcortical pathway underlying orienting gaze control is evolutionarily conserved (Dean et al., 1989; Kardamakis et al., 2016). This morphology makes “eye contact” a powerful means to communicate socially in typically developing people, whereas adults with ASD often report adverse emotional responses to looking eyes of others and avoid “eye contact” (Trevisan et al., 2017). It is hypothesized that the excitatory/inhibitory imbalance occurs in the subcortical visual pathway of those infants with ASD, leading to hyper-activation of the limbic system (Hadjikhani et al., 2018). The “eye contact” aversion would most likely develop at later juvenile/adolescent stages following human-unique processes. However, as noted above (Elsabbagh et al., 2013), those infants at risk for ASD show clear attention to faces as high as typically developing controls at 7–14 months of age, suggesting that the deficient attention is not necessarily linked with “eye contact” aversion in ASD.

#### 2.1.2 Biological motion

Biological motion (BM) preference comprises another aspect of the “CONSPEC” process; it is easily tested using highly reduced animations composed of relatively few light points (~ a dozen). Both human and chick neonates exhibit BM preference without visual experience (Simion et al. (2008) and Pavlova (2012) in human infants; Vallortigara et al. (2005) in chicks; also see Rugani et al. (2015) for the association of the BM preference with brain asymmetry). In addition to the commonality in their appearance at the early neonatal stage, both human infants and chicks show a clear inversion effect, that is, a preference for the upright walking motion over the inverted upside-down display [Troje and Westhoff (2006) in human infants; Vallortigara and Regolin (2006) and Chang and Troje (2009) in chicks]. Note also the recent report on the gravity prior, wherein naïve chicks preferred upward movements of single dot over the opposite downward (Bliss et al., 2023), in a manner similar to humans who judge upward motion more animate than the downward (Szego and Rutherford, 2008).

The motion characteristics of both local movements (such as the movements of the lower limbs) and global features (shape of the body) are critical in humans (Chang and Troje, 2009; Hirai et al., 2011). However, it remains unclear which of these visual components is associated with the development of social cognition. Bardi et al. (2011) reported that infants depend more on local cues, whereas Bidet-Ildei et al. (2014) highlighted the importance of translational displacement of the body. Hirai and Senju (2020) have proposed an integrative hypothesis of the two-process theory, wherein the “step detector” responsible for the local motions of feet below the body precedes the “bodily action evaluator” that processes the global processing of action types and styles. Several studies in both human adults and children have suggested the association between reduced sensitivity to BM and ASD (Blake et al., 2003; Rutherford et al., 2006; Wang et al., 2018; Kaliukhovich et al., 2021) [also see the recent meta-analysis (Federici et al., 2020)]. Importantly, it should be noted that, in our current context, visual preferences for social stimuli (face inversion, averted eye gaze, and BM) markedly differ between the two groups of infants with high and low familial risk of autism (Di Giorgio et al., 2016a). Further longitudinal studies are needed to determine the association between visual preference and the subsequent development of social interactions.

#### 2.1.3 Animacy

In addition to the face and BM, both chicks and humans have a visual predisposition to other cues of animacy (Rosa-Salva et al., 2011; Vallortigara and Rosa-Salva, 2017; Vallortigara, 2021b for reviews). For example, living organisms are characterized by self-propelling animacy, a well-established preference in both human babies (Di Giorgio et al., 2016b) and visually naïve chicks (Rosa-Salva et al., 2016, 2018). Unpredictable speed changes in motion are a critical feature of animacy in newborn human babies (Di Giorgio et al., 2021b), as observed in newly hatched chicks with distinct inherited variability (Versace et al., 2017, 2019) (for involvement of septal and hypothalamic nuclei, see Lorenzi et al., 2017). Both variability in body orientation and the unpredictable temporal contingency of motion are critical in chicks (Lemaire et al., 2022; Rosa-Salva et al., 2023). It should also be noted that avoidance of looking at (threatening) objects is also considered to be innately predisposed (Hebert et al., 2019).

#### 2.1.4 Imprinting and the early process of attachment formation

In chicks, the BM preference is functionally linked to filial imprinting. Imprinting is a complex process that involves predisposition and experience-based learning; thus, it may be homologous to the processes of attachment formation in human babies. Newly hatched domestic chicks and ducklings form lasting attachments, even when the first object seen is a non-biological artifact rather than a conspecific animal (Spalding, 1873; Lorenz, 1937). Artifacts such as rotating blue boxes were actually effective as imprinting objects (Horn, 2004). However, contrary to this popularly accepted idea, memorized preference for artifacts is short-lived and is gradually replaced by more naturalistic stimuli such as stuffed hens (Bolhuis et al., 1985; Johnson et al., 1985; Johnson and Horn, 1988). Accordingly, an innate predisposition gradually emerges after learned attachment fades. In contrast, BM preference emerges first and subsequently guides learning.

Perfectly naïve chicks show an apparent preference for BM, although with a relatively small effect size (Vallortigara et al., 2005). When imprinted by motion pictures, the BM preference is enhanced or “permissively induced” (Miura and Matsushima, 2012). The induction is nonspecific to the exposed stimulus, and any motion (even randomized point-light animation) is a similarly effective inducer. The memorized preference to the moving artifact, on the other hand, is determined by the visual features of the object such as color and shapes, thus is assumed to be “instructively induced.” However dissociable, the BM preference facilitates the memorized preference, or imprinting (Miura and Matsushima, 2016). BM animations were more effective than non-BM animations, and chicks with a higher BM preference exhibited higher imprinting scores.

**Figure 2:**
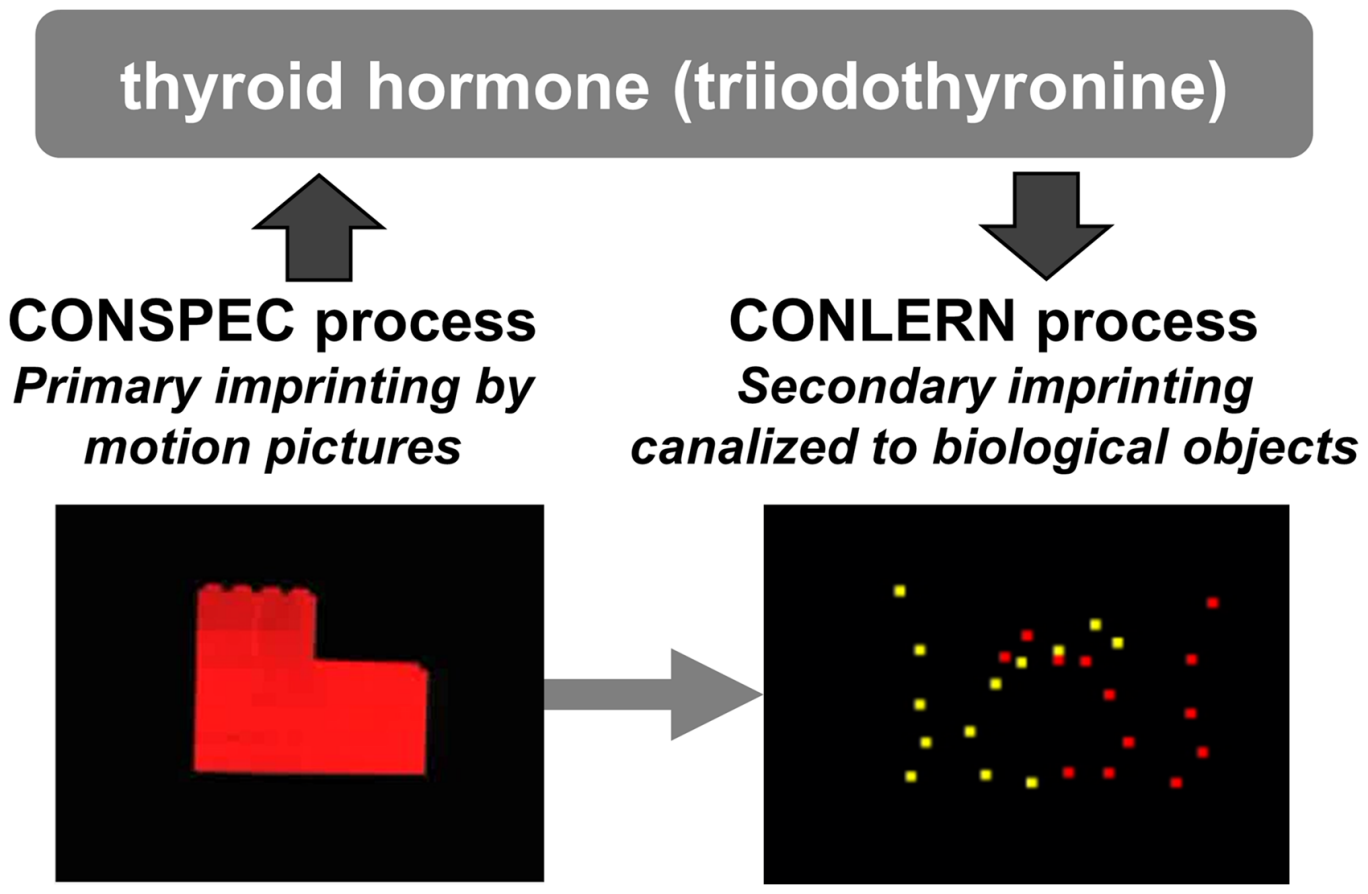
Thyroid hormone conversion in the central nervous system mediates the link between CONSPEC and CONLERN mechanisms toward adaptive socialization via visual canalization. Any motion pictures of artifact such as rotating red toy will effectively cause the primary imprinting selectively to the visual feature of the object. However, the primary imprinting is associated by CONSPEC mechanisms, wherein the biological motion (BM) preference is permissively induced if accompanied by enhanced *Dio2* gene expression and a rapid inflow of thyroid hormone (T_3_) in the dorsal pallium. Thyroid hormone subsequently elongates the sensitive period for memory formation, allowing the secondary imprinting for a longer post-hatch period. With the aid of the induced BM preference, the secondary imprinting is canalized to images of conspecifics, and the chicks will form firm social attachments to conspecifics.

Imprinting memory formation is functionally coupled with BM induction through enhanced thyroid hormone activity (Figure 2). Exposure to motion increases the expression of Dio2, which is responsible for the conversion of circulating thyroid hormone (T_4_) to its active form (T_3_) in the epithelial cells of telencephalic capillaries (Yamaguchi et al., 2012). The enhanced T_3_ influx into the dorsal pallium (intermediate medial mesopallium, IMM, an avian homolog of the mammalian neocortex, including the association areas) reopens the sensitive period and acutely strengthens learning and BM scores (Miura et al., 2018; Takemura et al., 2018). In aged chicks, T_3_ can reactivate the preference for animate objects (Lorenzi et al., 2021). Accordingly, imprinting allows chicks to remain imprintable for a prolonged period, guiding subsequent learning during the extended sensitive period to objects bearing BM features. Notably, these two aspects of imprinting (memory formation and induced predisposition) appeared tightly coupled and not dissociable (Miura et al., 2020).

Newly hatched domestic chicks could serve as a valid animal model for studying environmental risk factors for ASD, at least at the surface phenomena level. Imprinting is also assumed to be a primary stage of emotional development in human infants (Mobbs et al., 2016). The following section examines how ASD-like deficiencies could arise in the chick model and whether the underlying mechanisms are shared.

What are the predisposed priors for? Why should animals, and humans as well, be born knowing before birth (Vallortigara, 2021a)? A recent progress in machine learning could give us a hint (Barabási et al., 2023). Most artificial neural network models require extensive updating of network-wide weights among neurons by intensive training. Even though any randomly connected initial network could give rise to target functions after intensive and long training (Amari, 2020), learning processes are often slow and require intensive set of training examples, yet risk overfitting. By implementing genetic connectome model (GCM) (Kovács et al., 2020), the author showed that predisposed wiring should serve priors through evolutionarily shaped processes, thus promoting the computational power (Barabási et al., 2023). Genetic identities of neurons, as determined by the single-cell RNA seq in developing brains, could therefore be a powerful means to disentangle the molecular events responsible for the construct validity.

## 3 Construct validity

### 3.1 Valproic acid, an anticonvulsant drug, mediates ASD-like impairment of social behavior development and acute suppression of spontaneous embryonic movements

Given that VPA was identified as an environmental risk factor for ASD during pregnancy (Moore et al., 2000; Rasalam et al., 2005), studies have attempted to clarify the underlying mechanisms using rodent models [see reviews (Meador and Loring, 2013; Ergaz et al., 2016; Nicolini and Fahnestock, 2018)]. Late embryonic exposure to VPA impairs social behavior also in chicks (Nishigori et al., 2013; Sgadò et al., 2018; Lorenzi et al., 2019; Adiletta et al., 2021). These studies have consistently reported impaired social behavior in newborns and hatchlings after embryonic VPA exposure, although drug-induced phenotype disorders were not necessarily identical, probably due to task-dependent variation among individuals. For example, Matsushima et al. (2022) reported low imprinting scores exclusively in individuals with a low BM preference. Specification of the ASD-like behavioral impairments by the embryonic VPA awaits further studies.

Actually, VPA has a wide spectrum of pharmacological effects, including actions on N-methyl-D-aspartate type glutamate receptors [NMDA-R (Gean et al., 1994)] and inhibitory GABA transmission (Winterer, 2003). Moreover, VPA is well-accepted as a potent inhibitor of histone deacetylases [HDACs (Phiel et al., 2001)]. Acute anticonvulsant action on the late embryos (the last week of the prehatch development) may induce ASD-like phenotypes, as VPA effectively suppresses embryonic motion (Matsushima et al., 2022). Spontaneous motion is ubiquitous among late embryos of vertebrates (Bekoff et al., 1975) (see also Bekoff, 2001; Blankenship and Feller, 2010 for more recent reviews), although its functional roles remain unclear. Nevertheless, suppression of embryonic movements *per se* fails to account for ASD-like deficiencies in chicks, since similarly effective suppressers (e.g., selective blockers of NMDA-R MK-801) failed to cause ASD-like symptoms (Matsushima et al., 2022).

The brain regions and molecular events that are responsible for the VPA-mediated ASD phenotypes remain elusive. In a rat model, VPA enhanced NMDA-R expression and synaptic potentiation in the hippocampus (Rinaldi et al., 2007), thereby causing an imbalance between excitatory and inhibitory transmission (E-I imbalance; see Uzunova et al., 2016; Lee et al., 2017 for comprehensive reviews). In support of the hyper-excitation hypothesis, post-natal blockade of NMDA-R by memantine (a drug prescribed for Alzheimer’s disease) rescued social interaction impairment (Kang and Kim, 2016). Consistent with studies performed in rodent models, administering bumetanide (a selective blocker of NKCC1 co-transporter) immediately before training could rescue chicks with VPA-induced impaired imprinting (Matsushima et al., 2022); the impact of bumetanide will be discussed below.

### 3.2 Selective impairment of BM predisposition via embryonic interference with nAChR receptors, including neonicotinoid insecticides

Pesticide chemicals, particularly considering the rapidly increasing consumption of neonicotinoid insecticides [NNs (Costas-Ferreira and Faro, 2021)], are another serious concern in the etiology of ASD. NNs are designed to selectively perturb cholinergic neurotransmission in the nervous system of insects through their agonistic nature, whereas NNs were assumed to have low toxicity in vertebrates. Early ecological reports have highlighted the population decline of insectivorous birds (Hallmann et al., 2014). Following concerns regarding the high persistence of NNs in plants and soil, NNs were found to impair the migratory ability of granivorous birds (Eng et al., 2017, 2019). Several recent epidemiological studies have reported the risk of maternal exposure to environmental NNs (Keil et al., 2014; Gunier et al., 2017). An early study (Keil et al., 2014) estimated the association between the indoor usage of imidacloprid (IMI; one of the most heavily used NNs for flea and tick treatment for pet animals) and ASD, detecting an alarming odds ratio of ~2.0. Prenatal exposure to agricultural pesticides was found to be associated with low intelligence quotient and verbal comprehension (Gunier et al., 2017). A large-scale study on the association between ambient pesticide usage (NNs included) and ASD in California’s agricultural region (Von Ehrenstein et al., 2019) detected considerable odd ratios for various pesticide chemicals; the effects of prenatal exposure were boosted by additional exposure in neonatal infants. A rodent model study assessing acetamiprid (ACE; another NNs) has reported the abnormal development of social and anxiety-related behaviors in males after prenatal and lactational exposure (Ongono et al., 2020).

**Figure 3:**
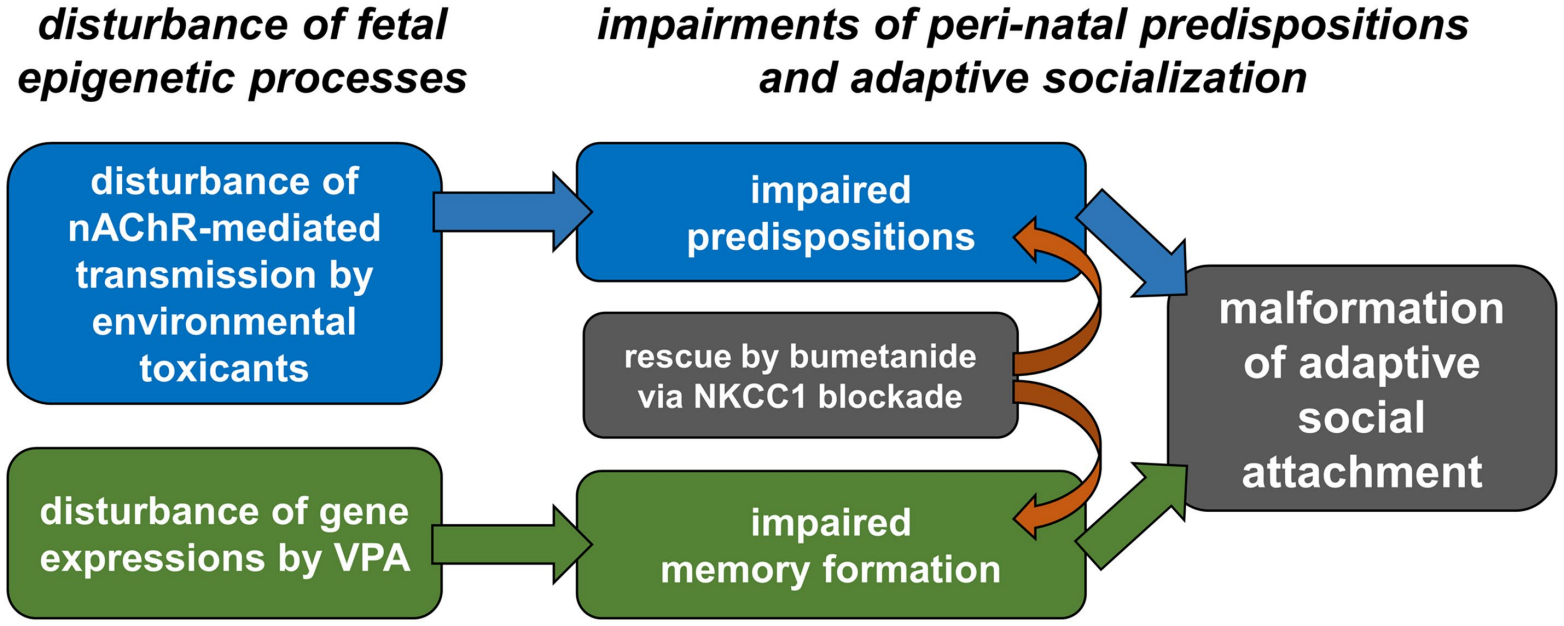
Disturbed epigenesis during the late embryonic stage leads to impaired predisposition critical for post-natal socialization through imprinting. Imidacloprid and other environmental toxicants disturbing nicotinic acetylcholine receptors (nAChR) would impair the induction of predisposed preference to biological motion. On the other hand, valproic acid (VPA) disturbs expression patterns of yet unspecified set of genes through inhibiting deacetylation of nuclear histone, leading to impaired memory formation. As both of the predisposition and the memory formation are critical for adaptive socialization, disturbance of either one of these would lead to social attachment hypoplasia. Despite distinct processes of disturbance, both impairments are rescued by bumetanide, a selective blocker of NKCC1 cotransporter.

Our study using a chick model (Sano et al., 2016) revealed high concordance with these reports in humans and rodents. Selective and non-selective blockade of nAChR (using tubocurarine and selective α7 subtype inhibitor), as well as perturbed nAChR transmission by IMI, could suppress embryonic movements and impair the BM preference of hatchlings. Notably, nAChR blockade did not impair imprinting memory formation, thus revealing distinct dimensions of social behavior malformation from those induced by VPA (Figure 3).

### 3.3 Thyroid hormone, E-I imbalance in humans and chicks

Maternal hypothyroidism (gestational hypothyroxinemia) is another risk factor for ASD (Román et al., 2013; Berbel et al., 2014; Getahun et al., 2018). Circulating levels of thyroid hormones (THs, T_4_ in particular) could be a potential early biological marker for ASD. To date, no consensus has been reached regarding the role of THs in the development of ASD, given that both positive (Hoshiko et al., 2011) and negative (Ames et al., 2020) results have been reported. In addition to being a critical determinant of imprinting in chicks (Lemaire et al., 2022), TH plays critical roles in diverse neurodevelopmental processes (Batista and Hensch, 2019), particularly in the maturation of GABAergic transmission via the rapamycin (mTOR; mechanistic target of rapamycin) cascade (Westerholz et al., 2013). The mTOR-GABA cascade may mediate the acute facilitatory effects of THs in chicks (Yamaguchi et al., 2012; Batista et al., 2018; Lorenzi et al., 2021). T_3_ acutely enhances GABAergic transmission in slice preparations of the chick pallium (Saheki et al., 2022), although its functional link to the behavioral effects remains elusive.

Interestingly, in humans, symptomatic autism comorbid with fragile X syndrome and tuberous sclerosis complex (TSC) is accompanied by mutations in the mTOR signaling pathway. Rapamycin was shown to rescue social impairment in a mouse model of TSC (Sato et al., 2012). A more recent study addressing macrocephaly in infants with ASD has suggested that synaptic pathology related to the mTOR pathway is responsible for hyperconnectivity (Pagani et al., 2021).

In the central nervous system, the metabolic control responsible for the balanced management of energy income and growth may underlie appropriate socialization during the early stages of life in humans and chicks. In addition, premature E-I balance (excitatory-inhibitory balance, or delayed GABA switch from depolarizing to hyperpolarizing response) could be a key event in neural maturation, affording a potential target for developing effective pharmacotherapies for ASD.

### 3.4 GABA switch, nicotinic transmission, and treatment using bumetanide and oxytocin

GABA exerts depolarizing transmission via GABA-A receptors in the embryonic and early post-natal stages, and the excitatory GABA is supposed to exert trophic functions for the functional maturation of the brain. During the perinatal period, excitation is converted to adult-type inhibitory neurotransmission [Ben-Ari (2002), Represa and Ben-Ari (2005), and Ben-Ari et al. (2007) for comprehensive reviews; Pfeffer et al. (2009) for the hippocampal development; also see Hyde et al. (2011) specifically for the schizophrenia etiology]; this conversion (referred to as the GABA switch) is mediated by a reduction in the intracellular chloride ion ([Cl^−^]_i_), which, in turn, can be attributed to the enhanced expression of cation-linked co-transporters responsible for the efflux of Cl^−^ (KCC2 over NKCC1). To identify regulatory mechanisms underlying the GABA switch, nicotinic cholinergic transmission was found to be critical in the chick ciliary ganglion (Liu et al., 2006). Further analysis of the phosphorylation of KCC2 molecules revealed protein kinase C-mediated modulation by glutamatergic and serotonergic actions, as well as the activation of muscarinic acetylcholine receptors (Kahle et al., 2013) (for more recent comprehensive reviews see Kaila et al., 2014; Zhang et al., 2021). Cascade of causal molecular events is yet unspecified.

The association between GABA switch retardation and ASD was based on the finding that diazepam (an anxiolytic drug, a positive modulator of GABA-mediated inhibition used to relieve anxiety) could paradoxically increase aggressive behaviors in autistic children (Marrosu et al., 1987). Subsequent studies have shown that dysfunctional GABA inhibition could play a pivotal role in ASD etiology (Nelson and Valakh, 2015; Hadjikhani et al., 2018). Bumetanide has attracted attention as a potent candidate for ameliorating ASD symptoms, given that this agent selectively blocks the NKCC1 co-transporter responsible for Cl^−^ influx (Delpire and Ben-Ari, 2022). Although initial open-label small-sized trials appeared positive and promising (Fernell et al., 2021; Wang et al., 2021), recent phase-2 trials failed to afford positive outcomes (Sprengers et al., 2021). A detailed follow-up analysis has identified heterogeneous phenotypes of neurocognitive impairment in patients with ASD, some of which were unaffected by bumetanide (Van Andel et al., 2023). In a chick model, bumetanide treatment immediately before training rescued impairments in both imprinting (by VPA) and BM preference (by nAChR blockade) (Matsushima et al., 2022). Further studies are required to identify underlying targets and pharmacology.

Oxytocin and related nonapeptides comprise another group of candidate drugs for ASD. In humans, intranasal application of oxytocin can acutely ameliorate social deficiencies such as BM perception and social communication in cases of relatively low severity (Kéri and Benedek, 2009; Parker et al., 2017). In typically developing individuals, oxytocin receptors peak during early childhood, whereas this peak is absent in those with ASD (Freeman et al., 2018). Similar facilitatory effects of oxytocin on prosocial behaviors have been observed in dogs (Nagasawa et al., 2015; Kovács et al., 2016), birds (Duque et al., 2018; Loveland et al., 2019; Seguchi et al., 2022) and fish (Nunes et al., 2020), suggesting its ubiquitous role in vertebrates. Considering the underlying mechanisms, a study using oxytocin-receptor knock-out mice has suggested that KCC2 is regulated by oxytocin (Leonzino et al., 2016). A more recent study in mice identified a link between the genetic risk of ASD and the oxytocinergic signaling pathway (Hörnberg et al., 2020). In chicks, intracranially administered mesotocin (an avian counterpart of oxytocin) enhanced the preference of naïve chicks (Loveland et al., 2019). The appropriately timed excitation/inhibition balance could play critical roles on functional maturation of the social brain network in both of the newly hatched chicks and the human neonates, thus forming another aspect of the convergent peri-natal “bottleneck” (Figure 1).

## 4 Perspectives

Do chick studies have future? Table 1 summarizes the validity and limitations of chicks as an ASD model animal, and some of these points will be discussed in detail. To conclude this review article, we will further discuss how neurocognitive homologies are to be defined. We will also discuss how we should address the homology issue on firm biological bases.

**Table 1:** Validity, advantages and limitations of the newly hatched domestic chicks as an ASD model animal.

Surface validity
Predisposed priors of social communications shared with humans. Preference to face configuration. Preference to biological motion (BM). Preference to animacy. Two processes of CONSPEC and CONLERN.
Construct and predictive validity
Cellular/molecular mechanisms shared with humans. Effects of valproic acid in the late embryonic stage. Effects of neurotransmission by nAChR in the late embryonic stage. Effects of hypothyroidism. Excitatory-inhibitory imbalance (GABA-A reversal potential). Effective treatment of neonates by bumetanide.
Advantages of the chick model
Tractability of fertilized eggs and hatchlings. Availability of embryonic manipulation. Rapid testing after pharmacological treatment of late embryos. No confounding effects of maternal metabolism. Functional “subcortical” visual pathway. Accumulated literatures on the development. The principles of the 3Rs. Replacing mammalian models. Reduction due to reproducible results with relatively few subjects. Refinement due to availability of neurocognitive tests in neonates.
Disadvantage and limitations of the chick model
Taxonomic difference from *Homo sapiens*. Distinct behavioral development in juvenile/adults. Lack of layered isocortex in telencephalon. Lack of pure lines for studying genetic bases of behavioral development. Lack of powerful methods for genetic manipulations. Lack of data on verbal communication and vocalization. Lack of data on restricted and repetitive patterns of behavior.

### 4.1 Limitations and advantages of the domestic chick; comparisons with other novel ASD model animals

In terms of the taxonomic validity measured by the distance from *Homo sapiens* (Belzung and Lemoine, 2011), chicks are definitely inferior model of human disorders because they are not mammals. Non-human primates such as common marmosets (Watanabe et al., 2021) is one of the most promising options in this respect. Prenatal VPA treatment actually causes a deficient inequity aversion (Yasue et al., 2018), suggesting impaired social motivation or interest to conspecifics. Although marmosets have the ability to visually perceive BM (Brown et al., 2010), effects of prenatal VPA treatment have not been addressed in the neonatal visual perception. Genetic engineering technique using the CRISPR/Cas9 system has successfully generated marmosets that carry mutations in the fragile X mental retardation 1 (FMR1) (Abe et al., 2021). Though fragmental at present, these studies are expected to yield a promising model system, high in both face and construct validity in near future.

Zebrafish is another promising model to study ASD despite the inferior homological validity (Choi et al., 2021). Several technical advantages such as the large-scale pool of mutants, wide applicability of tractable genetic manipulation and high-resolution whole brain imaging, makes this fish a powerful candidate for studying the molecular basis of developmental disorders including ASD (Geng and Peterson, 2019; Torres-Pérez et al., 2023). High throughput screening of the environmental risk chemicals (Geng et al., 2022) is probably the most successful product so far.

Comparison with these two models clearly reveals limitations and advantages of domestic chicks as ASD model. As the genetic engineering is premature in birds, the chick model is inappropriate for experimental manipulation of relevant genes. The rodent models are superior to specify the genes involved in the ASD etiology. On the other hand, environmental risk chemicals are reliably searched for in chicks and fish because (1) chemicals can be quantitatively applied to eggs and (2) rapid development allows efficient screening. Furthermore, (3) complications due to maternal metabolisms are inevitable in mammals, while these are disregarded in studies using chick and fish models. The screening efficiency is much higher in fish than any other models, however, the marmoset and chick models are superior in their behavioral similarity to humans. To examine the BM predisposition, for example, similar video clips of point light animation are used for testing chicks (Vallortigara et al., 2005), marmosets (Brown et al., 2010) and human babies (Simion et al., 2008), whereas motion pictures of different kinetics are used in zebrafish (Larsch and Baier, 2018).

### 4.2 Is the similar social perception due to genuine homology or convergent evolution?

The construct validity of chicks as the ASD model could be assumed to be evidence in favor of the homology, however, the present list is inevitably not exhaustive. ASD is actually a spectrum composed of a variety of diverse phenotypes among individuals with different genetic and environmental backgrounds. Any animal model would therefore be partial, covering limited aspects of the disorder. It is therefore critically important to collect any pieces of disproof, namely the discrepancy between the model and the human cases in their etiology, and also between the model animals of different taxa, such as rodents, primates, birds and fish.

So far, two major points remain ambiguous, making the answer to this question difficult. One is to specify the responsible brain region for the priors, and another is the molecular events leading to the formation of the predisposed social perception. As for the first, we tentatively assume the subcortical visual pathway, however, its development during the late embryonic stages is largely unknown. For the second, as would be expected from the VPA effects, a variety of epigenetic processes are supposed to underlie, such as DNA methylation, histone modification and post-translational splicing (Eshraghi et al., 2018; Leung et al., 2023). It is also noticed that the phylotypic bottleneck of the vertebrate morphogenesis is characterized by a transition in chromatic accessibility (Uesaka et al., 2019). Comprehensive survey is expected on the developmental changes of chromatin environment in embryos of these assumed model animals, particularly in its late stage of development.

Another aspect could be the blood–brain-barrier (BBB), because premature BBB of the embryonic brains could be responsible for the fragility to environmental toxicants, yet some of the BBB functions are conserved among vertebrates and even some invertebrate animals (O’Brown et al., 2018). At the same time, the endothelial epithelial cells of blood vessels in the brain are actively regulating the endocrine control, such as thyroid hormone in chicks (Yamaguchi et al., 2012) and juvenile hormone for cast differentiation in ants (Ju et al., 2023); BBB could play as an active regulator or “gatekeeper” of the humoral factors in the developing CNS, rather than immature underdeveloped barriers. In this context, epigenetic regulation of BBB in healthy and damaged brain needs more attention (Ihezie et al., 2021); for example, histone deacetylase inhibitor such as VPA (see above) could protect the BBB damages after stroke. Possible links connecting between BBB development and ASD etiology are to be searched for in future, with a close reference on the involvement of gut-brain axis in ASD (Morton et al., 2023).

## 5 Concluding remark

No single animal models suffice to understand the complex etiology, cellular/molecular mechanisms, responsible brain networks, behavioral traits and pharmacological therapeutics of ASD. Though inevitably fragmental, findings obtained from a variety of, but appropriately chosen animal models should be complementarily integrated for a proper understanding of a developmental disease with multiple causes and wide phenotypic spectrum. Developmental homology to human neonates would be most critical criteria of the valid animal models. In this respect, the domestic chick comprises a unique position for studying neurocognitive disorders.

A fundamental question remains. Why did chicks converge to human neonates despite distinct evolutionary separations over 300 million years? This remains an unresolved puzzle, which could be addressed by analyzing behavioral phenotypes and disorders (surface validity) and the underlying mechanisms (construct validity). Biological psychiatry and comparative psychology therefore ask a same question: *What are we?*

## Author contributions

TM: Conceptualization, Funding acquisition, Investigation, Writing – original draft. TI: Conceptualization, Supervision, Writing – review & editing. GV: Conceptualization, Funding acquisition, Supervision, Writing – review & editing.
